# Topic Modeling of Nursing Issues in the Media During 4 Emerging Infectious Disease Epidemics in South Korea: Descriptive Analysis

**DOI:** 10.2196/60446

**Published:** 2025-01-06

**Authors:** Jungok Kim, Eun Kyoung Yun

**Affiliations:** 1 College of Nursing Science Kyung Hee University Seoul Republic of Korea

**Keywords:** topic modeling, news articles, nursing issues, text analysis, emerging infectious disease

## Abstract

**Background:**

Emerging infectious disease disasters receive extensive media coverage and public attention. Nurse burnout and attrition peak during health crises such as pandemics. However, there is limited research on nursing issues related to repeated emerging infectious disease crises over time.

**Objective:**

The purpose of this study was to analyze and draw implications from changes in key nursing issues reported by the news media during the outbreaks of severe acute respiratory syndrome (SARS; 2003), influenza A (2009), Middle East respiratory syndrome (MERS; 2015), and COVID-19 (2020) in Korea using topic modeling.

**Methods:**

A total of 51,489 news articles were extracted by searching for the keywords “nursing” or “nurse” in the title or body of articles published from April 2003 to May 2021 (during new infectious disease outbreaks) in the open integrated database. The selected news articles were preprocessed then analyzed for text and structure using a 3-step keyword analysis method, latent Dirichlet allocation topic modeling, and keyword network analysis.

**Results:**

Among the 51,489 news articles collected with the search terms “nursing” and “nurse,” 17,285 (33.6%) were selected based on the eligibility criteria and used in the final analysis. Using topic modeling, we derived 5 topics each for SARS, influenza A, and MERS and 6 topics for COVID-19. The themes commonly identified through topic modeling and keyword network analysis across the 4 epidemics were “response to emerging infectious diseases in Korea,” “demand for nurses,” “vulnerability in the work environment,” and “roles and responsibilities of nurses.” Although the topic names were the same, the meanings implied by the comprehensive keywords for each epidemic varied depending on the epidemic and the times.

**Conclusions:**

Analysis of the identified themes and associated keyword network revealed that issues related to nurse shortages, working conditions, and poor treatment were not unique to the COVID-19 pandemic but rather recurring themes from previous epidemics. Our findings can be used to inform strategies to improve the professional roles, work environment, and treatment of nurses during health crises. Suggestions for future nursing-related policy impact and change research are also provided.

## Introduction

An emerging infectious disease (EID) is a newly recognized or previously known disease characterized by new virulence or spread in previously unaffected areas [1]. Since the 2000s, South Korea has had 4 EIDs: severe acute respiratory syndrome (SARS) in 2003, novel swine-origin influenza A (H1N1) in 2009, Middle East respiratory syndrome (MERS) in 2015, and COVID-19 in 2020. Among them, influenza A and COVID-19 were declared as pandemics by the World Health Organization (WHO). Antiviral treatments were provided free of charge in Korea starting in August 2009 during the SARS outbreak, after which the number of infections and deaths were no longer regularly recorded. Before then, 270 deaths and 2417 infections were recorded, and at the time of manuscript submission, SARS had resulted in an additional 0 deaths and 4 infections according to official reports. Although the exact number of infections has not been counted since August 2009, more than 700,000 cases had been prescribed antiviral drugs from August 2009 to December 2009. During the MERS period, there were 39 deaths and 186 infections. Regarding COVID-19, 34,572,552 confirmed cases and 35,605 deaths were reported from January 20, 2020, to August 30, 2023, the period involving full monitoring [2].

EID catastrophes receive extensive media coverage and public attention. Indeed, public interest in nurses peaks during health crises, such as infectious disease epidemics [3]. EID outbreaks increase public recognition of and attention to the dedication of nurses working on the front lines of the disaster response [4]. Meanwhile, nurses experience physical and psychological burnout as well as professional ethical conflicts owing to the risk of infection, heavy workloads, patient violence, and the stigma of being a carrier of infection [5-8]. Rising rates of nurse burnout and resignation during inadequate infectious disease crisis responses have become global concerns [9,10]. The loss of skilled nurses may lead to a decline in the quality of nursing care and organizational nursing capacity, which may pose a risk to national health crisis responses and public health.

Although infectious disease outbreaks can be traumatic for individuals and communities, they can also lay the groundwork for health system transitions after the outbreak ends, through institutional and policy improvements that identify and address health system weaknesses revealed during the response. With respect to issues in the nursing workforce, scholars have suggested that societal consensus for improvements in working conditions and institutional arrangements for health resources and protection are needed to minimize nurse burnout and attrition in special situations, such as infectious disease outbreaks [6,8,11,12]. At the national level, discussions about appropriate investments and compensation systems for the training and deployment of infectious disease professionals and collaboration with the medical community are needed, whereas at the societal level, increasing public awareness of health workers and building public consensus through media coverage have been emphasized as obligations [7,13,14]. Media’s reports of issues in the field of infectious diseases play an important role in forming society’s perceptions of an infectious disease response, nursing, and nurses. Problems that are not resolved over a long period of time, occur repeatedly, and increase public interest become social issues, which become part of a public agenda that the general public recognizes as appropriate for the government to solve. This public agenda is then developed and set as a specific policy agenda.

Previous studies analyzing media coverage of infectious disease outbreaks focused on public perceptions of nursing portrayals and issues [3,4,15,16] and were mostly limited to specific epidemic periods. However, these studies did not track how nursing issues were portrayed during recurrent outbreaks since the 2000s, the unique characteristics of each period, and how characteristics have changed. In the context of COVID-19, which was declared as an endemic by the WHO, policies that address the underlying issues and promote a better future urgently need to be developed. Prior studies using content analysis techniques to analyze data collected through standardized questionnaires or media inevitably had limited data to reflect public perceptions and trends. To compensate for this, it is necessary to grasp the meaning inherent in big data such as online news and social media, and topic modeling and keyword network analysis methods, which have been widely used recently to analyze unstructured text in big data, overcome the limitations of manual analysis by humans [17-19].

Therefore, this study aimed to analyze the changes in major nursing issues reported by the news media when 4 new infectious diseases occurred in the 2000s using topic modeling and keyword network analysis methods and derive the implications. Specifically, we aimed to identify the issues that should be considered to effectively respond to new infectious disease crises that may occur in the future and ensure public safety. Our findings could provide the basis for the development of desirable infectious disease response strategies.

## Methods

### Data Extraction

We collected data from the Korea Press Foundation’s article information website [20], an open-access database containing news articles from 54 media organizations. We collected data published between April 2003 and May 2021 corresponding to the periods of EID epidemics. Specifically, we analyzed news articles published during the SARS, influenza A, MERS, and COVID-19 outbreaks.

**Figure 1:**
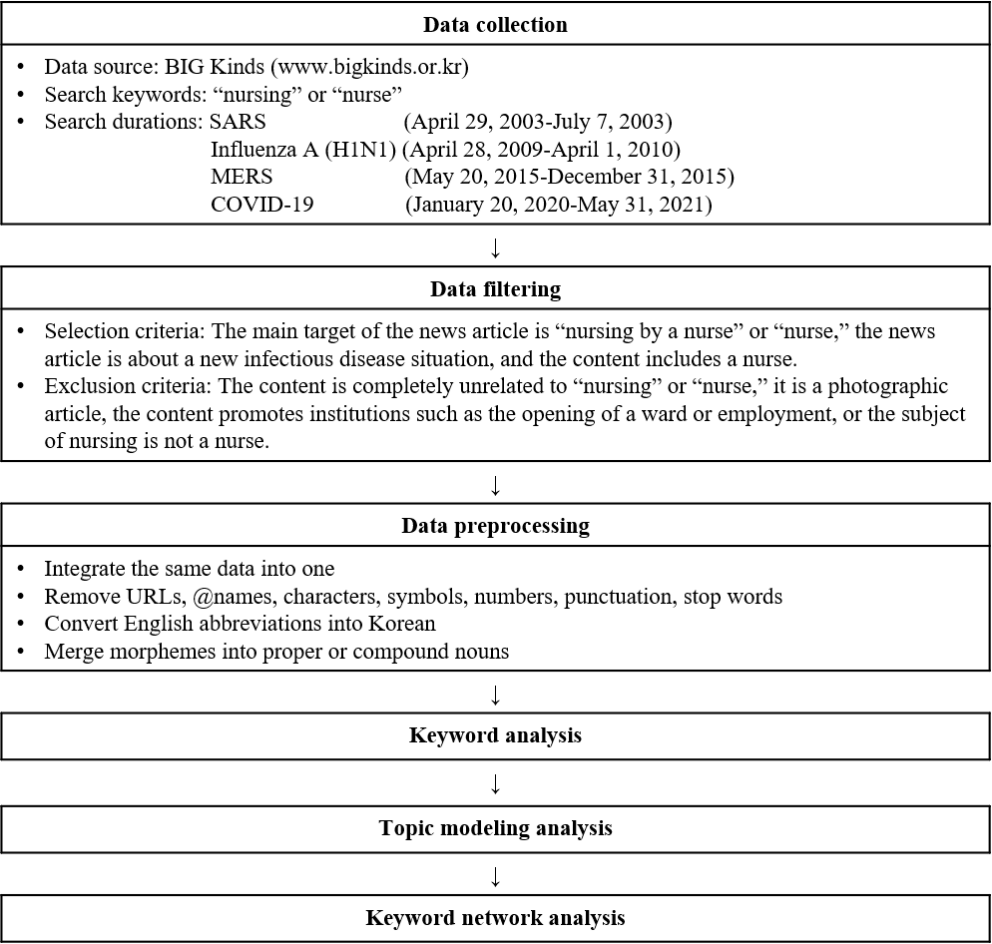
Study process. MERS: Middle Eastern respiratory syndrome; SARS: severe acute respiratory syndrome.

A total of 51,489 news articles were extracted by searching for “nursing” or “nurse” as the keyword in the title or text of the article during the outbreak of each new infectious disease. Although the terms “nursing” and “nurse” appeared in the text of these articles, irrelevant content, such as promotional material, was excluded using the selection and removal criteria detailed in Figure 1. These criteria were informed by advice from experts in the field of nursing research and clinical experience. A total of 17,285 news articles were selected using these criteria and used for the final analysis. Figure 1 details the data extraction process used in this study.

### Data Preprocessing

We preprocessed the metadata of the selected news articles using the *KoNLP* and *tm* packages for natural language processing in R version 4.1.0. Specifically, we removed terms, including symbols, special characters, names of private individuals, and media company names. English terms, such as “SARS” and “OECD,” were occasionally removed during terminology preprocessing; these terms were converted into their Korean equivalents to maintain consistency. During morpheme analysis, complex nouns like “living treatment center” were segmented into “living,” “treatment,” and “center,” causing loss of meaning. To address this, these terms were reconstituted as proper or compound nouns based on N-gram analysis and the context of the original article. Abbreviated Korean terms were replaced with their official names to ensure terminological consistency**.**

### Keyword Analysis

We used the *tm* package in R version 4.1.0 to calculate word frequencies and determine the importance of specific terms using the term frequency-inverse document frequency metric value. Statistical weights were calculated to assess the significance of specific terms within the corpus for keyword analysis of the news articles.

### Topic Modeling

Topic modeling classifies and integrates topics in extensive document sets using probabilistic statistical methods to identify latent high-level concepts, extract the topics implicit in the documents, and derive common high-level concepts latent in the topics [21]. We used the latent Dirichlet allocation (LDA) algorithm of the *topicmodels* package of R version 4.1.0 to analyze the topics embedded in the documents. The coding of the analysis was based on the work of Grün and Hornik [16] and open-source code on the GitHub site.

We configured the LDA algorithm by setting the document extraction parameter (α) to 50/k, and the previous parameter delta (or eta) of the word distribution constituting the topic was set to 0.1. Gibbs sampling was used to estimate the topic distribution for the document, with 1000 repetitions used for Gibbs sampling [16,22].

We also conducted a quantitative analysis to select the appropriate number of topics. We calculated perplexity values ranging from 2 to 20 using the *topic models* and *LDAvis* packages of the R program. The topic modeling analysis was repeated by applying different numbers of topics based on the perplexity values. For the analysis, we repeated the adjustment of the λ value of the LDA visualization and reviewed the original text of the news articles to extract a list of the 30 most representative keywords for each topic.

### Keyword Network Analysis

Keyword network analysis visualizes keyword relationships by calculating co-occurrence frequencies and metrics such as connection centrality, proximity centrality, and mediation centrality [23]. We performed keyword network analysis using the *Network*, *sna*, and *ggplot2* packages in the R program to improve the specific content and structure of the topics and the words constituting the topics derived from thematic modeling. Considering the visibility of the network around thematic keywords, the correlation coefficient was established, and the relationship between the simultaneous appearance of words was analyzed.

The top 15 keywords were selected as the main keywords for each topic through quantitative analyses including topic modeling analysis, keyword network analysis, and verification by nursing experts, and the topic names reflected the same higher-level concept to examine the trends of the topics and keywords that make up the topic by period of the new infectious disease based on the main keywords. In this study, 15 keywords for the new infectious disease topic and topic are presented.

### Ethical Considerations

All news article data analyzed in this study were publicly available and not subject to copyright restrictions. The Institutional Review Board of Kyung Hee University approved this study (IRB number KHSIRB-21-334(EA)).

## Results

### News and Keyword Counts

After preprocessing the data from a total of 17,285 news articles, we extracted 1974 words for the SARS epidemic, 9520 words for influenza A, 15,639 words for MERS, and 24,888 words for COVID-19, resulting in a total of 52,021 words. The numbers of news articles and keyword types analyzed increased progressively across successive infectious disease outbreaks, and the average daily number of news articles was the highest during the MERS period.

### Top Topics and Keywords During Epidemics

Table 1 presents the keywords and topic names identified for each epidemic period. We selected 5 topics each for SARS (perplexity=1599.241, λ=0.7), influenza A (perplexity=977.101, λ = 0.8), and MERS (perplexity=1717.432, λ=0.7) and 6 topics for COVID-19 (perplexity=2515.911, λ=0.6). We sorted the topics in the order of the largest percentage of word clusters that make up the topic and identified them as themes.

**Table 1:** Themes and keywords of the topics.

Themes and topics			Top keywords contributing to the topic model		Token, %
**Theme 1: Response to emerging infectious diseases in Korea**					
	SARS^a^ (Topic 3)	SARS, patient, hospital bed, infection, isolation, disinfection, suspected patient, outbreak, designation, health personnel, presumed patient, National Institute of health, constitute, dedication, countermeasure		18.2	
	Influenza A (H1N1; Topic 2)	Influenza A virus(H1N1), infection, hub-hospital, spread, outbreak, prevention, medical staff, treatment, mask, test, suspected case, confirmed case, countermeasure, the dead, Tamiflu		21.7	
	MERS^b^ (Topic 3)	MERS, confirmed case, infection, addition, medical staff, hospital, emergency room, treatment, exposed, isolation, Central MERS Control Countermeasure Headquarters, closed, S-hospital^c^, K-hospital^c^, P-hospital^c^		21.5	
	COVID-19 (Topic 2)	COVID-19, medical staff, mask, protective clothing, world, treatment, China, pandemic, virus, outbreak, hospital room, situation, the dead, hospitalization, pneumonia		20.8	
**Theme 2:** **Demand for nurses**					
	SARS (Topic 1)	The aged, nursing care, society, nurse, facility, family, role, profession, psychiatric, system, push ahead, policy, dementia, the disabled, welfare		31.2	
	Influenza A (H1N1; Topic 1)	Nurse, nursing college, education, service, push ahead, nursing department, the aged, establishment, system, manpower, expansion, employment, quota, the disabled, insufficiency		26.3	
	MERS (Topic 1)	Expansion, comprehensive nursing service, push ahead, countermeasure, hospital, provide, nursing staff, system, infection disease, caregiving, nurse’s aide, measure, reinforce, improvement, nationwide		23.6	
	COVID-19 (Topic 3)	Securing, support, treatment, medical worker, insufficiency, deploy, dispatch, nurse, critical patient, Daegu, living treatment center, response, allowance, recruitment, dedicated hospital		17.7	
**Theme 3: Vulnerability in the working environment**					
	SARS (Topic 2)	Son and daughter, woman, child, risk, exercise, person, workplace, night duty, trust, health, nurse, research, thought, parent, prevention		19.7	
	Influenza A (H1N1; Topic 4)	Doctor, nurse, patient, medical, hospital bed, OECD^d^, level, medical service, facility, medical expenses, Korea, region, insufficiency, intensive care unit, increase		18.7	
	MERS (Topic 5)	MERS, isolation, patient, suspicion, contact, action, duty, hospital, Korea Centers for Disease Control and Prevention, nurse, infection, test result, infected person, addition, emergency room		16	
	COVID-19 (Topic 4)	Confirmed case, infection, nurse, hospital, group infection, duty, contact, convalescent hospital, screening center, rest, emergency room, working environment, site, manpower, protective clothing		17.5	
**Theme 4: Roles and responsibilities of nursing professionals**					
	SARS (Topic 5)	Nurse, hospital, suspicion, blood, doctor, use, uncover, sexual harassment, nurse’s aide, investigation, contravention, drunk driving, illegality, measurement, injection		15.4	
	Influenza A (H1N1; Topic 3)	Hospital, nurse, doctor, treatment, family, death, incident, contravene, criminal investigation, grandmother, death with dignity, obstetrics and gynecology, adjudge, false, infringement		21.1	
	MERS (Topic 2)	Hospital, medical staff, nurse, doctor, treatment, family, thought, person, heart, intensive care unit, child, oneself, protective clothing, gratitude, cheer		21.8	
	COVID-19 (Topic 1)	Doctor, government, Korean Medical Association, expansion, policy, push ahead, national assembly, strike, region, improvement, medical resident, Korean Nursing Association, the public, medical school quota, found		23.9	
**Theme 5: Government response and public opinion**					
	MERS (Topic 4)	MERS, situation, virus, the public, outbreak, symptom, response, fear, transmission, government, information, preventive measures, open, anxiety, the Middle East		17	
	COVID-19 (Topic 5)	Nurse, president, the public, gratitude, heart, commitment, encouragement, message, social network service, cheer, consolation, controversy, criticism, expression, labor		13	
**Theme 6: Vaccination for emerging infectious diseases**					
	Influenza A (H1N1; Topic 5)	Vaccination, student, influenza A virus(H1N1), school, vaccination, public health center, start, doctor, nurse, constitute, school nurse, plan, securing, school parent, insufficiency		12.2	
	COVID-19 (Topic 6)	Vaccine, inoculation, AstraZeneca, injection, Korea Disease Control and Prevention Agency, start, public health center, side effect, Pfizer, effect, nurse, nursing facility, targets, worker, launch		7.1	
**Theme 7: Response to emerging infectious diseases in other countries**					
	SARS (Topic 4)	SARS, China, the dead, Taiwan, spread, outbreak, WHO^e^, Hong Kong, Singapore, infected person, death, Canada, Vietnam, transmission		15.5	

^a^SARS: severe acute respiratory syndrome.

^b^MERS: Middle East respiratory syndrome.

^c^Abbreviations for specific hospital names.

^d^OECD: Organisation for Economic Co-operation and Development.

^e^WHO: World Health Organization.

### Trends in Nursing-Related Topics in Media Coverage

According to the topic modeling analysis, we classified a total of 7 topics from all news articles, and the topics commonly derived from the 4 infectious disease periods were “response to emerging infectious diseases in Korea,” “demand for nurses,” “vulnerability in the working environment,” and “roles and responsibilities of nursing professionals.” Although the overarching concepts remained consistent, the keywords comprising each topic varied across the duration of infectious diseases. Notably, the keywords that comprised the topics for each epidemic period differed. We observed that the topic “vaccination for emerging infectious diseases” appeared only in the influenza A and COVID-19 pandemics, and “government response and public opinion” appeared only in the MERS and COVID-19 periods. Moreover, the topic “response to emerging infectious diseases in other countries” appeared only during SARS (Table 2). Table 1 shows the topics and word clusters identified by our model.

**Table 2:** Trends in nursing-related topics in media coverage, reported as tokens for each outbreak.

Trends	SARS^a^, %	Influenza A (H1N1), %	MERS^b^, %	COVID-19, %
Response to emerging infectious diseases in Korea	18.2	21.7	21.5	20.8
Demand for nurses	31.2	26.3	23.6	17.7
Vulnerability in the working environment	19.7	18.7	16	17.5
Roles and responsibilities of nursing professionals	15.4	21.1	21.8	23.9
Government response and public opinion	—^c^	—	17.1	13
Vaccination for emerging infectious diseases	—	12.2	—	7.1
Response to emerging infectious diseases in other countries	15.5	—	—	—

^a^SARS: severe acute respiratory syndrome.

^b^MERS: Middle East respiratory syndrome.

^c^Not applicable.

### Theme 1. Response to Emerging Infectious Diseases in Korea

During the 4 periods, the “response to emerging infectious diseases in Korea” topic accounted for approximately 20%, ranking third during the SARS and MERS outbreaks and second during the influenza A and COVID-19 outbreaks. The keyword patterns were similar between SARS and MERS and between influenza A and COVID-19, reflecting trends observed in the keyword frequency analysis.

Keywords for each period include the name of the respective virus and terms describing the outbreak situation, such as “infection,” “suspected patient,” “outbreak,” and “presumed patient” during the SARS period; “infection,” “spread,” “outbreak,” “confirmed patient,” and “deaths” during the influenza A period; “confirmed case,” “infection,” “additional,” “quarantine,” and “closure” during the MERS period; and “China,” “pandemic,” “outbreak,” “situation,” “world,” and “the dead” during the COVID-19 period, indicating the evolving scale and severity of infectious disease outbreaks in Korea over time.

In addition, keywords related to infectious disease response and preparedness such as “isolation,” “disinfection,” “designation,” “National Institute of Health,” “constitute,” and “dedication” during SARS changed to “hub-hospital,” “prevention,” “treatment,” “mask,” “countermeasure,” “test,” and “Tamiflu” during the influenza A and MERS periods and keywords related to preparation such as specific hospital names and “hospital,” “emergency room,” “Central MERS Control Countermeasure Headquarters,” “treatment,” “isolation,” and “closed” during the COVID-19 period. Keywords related to preparation, such as “mask,” “protective clothing,” “treatment,” and “hospitalization” diminished in number, with response-focused keywords emerging in their place.

### Theme 2. Demand for Nurses

The topic “demand for nurses” ranked first and represented the largest proportion during the SARS to MERS periods; during COVID-19, the topics of “roles and responsibilities of nursing professionals” and “response to emerging infectious diseases in Korea” represented higher proportions, with “demand for nurses” dropping to third place. When examining the keyword flow of topics by period, the keywords “system,” “policy,” “countermeasure,” and “response” were associated with “the aged,” “society,” “facility,” “psychiatric,” “dementia,” “the disabled,” and “welfare” during the SARS period; “service,” “the aged,” and “the disabled” during the influenza A period; “comprehensive nursing service,” “hospital,” and “infection disease” during the MERS period; and “Daegu,” “living treatment center,” and “dedicated hospital” during the COVID-19 period, showing that the areas in which nurses were needed and the reasons for a nursing demand changed as a result of health and welfare systems and policies for infectious disease responses.

In addition, “nursing care,” “family,” “profession,” and “role” during the SARS period; “manpower” during the influenza A period; “nursing staff,” “caregiving,” and “nurse’s aide” during the MERS period; and “treatment,” “medical worker,” and “critical patient” during the COVID-19 period represent keywords related to the perception of the role of nurses. The keywords “nursing college,” “nursing department,” “expansion,” and “quota” during the influenza A period; “nurse’s aide” during the MERS period; and “support,” “deploy,” “dispatch,” “allowance,” and “recruitment” during the COVID-19 period represented changes in ways to meet the nursing demand by period.

### Theme 3. Vulnerability in the Working Environment

The topic “vulnerability of the working environment” had the second highest weight during the SARS period, but its weight decreased during the influenza A and MERS periods. Its weight increased to 4th place during the COVID-19 period. During the SARS period, keywords such as “son and daughter,” “woman,” “parent,” “workplace,” “thought,” “risk,” “exercise,” “night duty,” “health,” and “prevention” were topics about women’s conflicts between work and childcare and health risks related to work, while during the influenza A period, keywords such as “patient,” “hospital bed,” “OECD,” “level,” “facility,” “insufficiency,” “intensive care unit,” and “region” showed a quantitative shortage of nurses and medical facilities. During the MERS period, news reports focused on topics such as “contact,” “infection,” “addition,” “duty,” and “emergency room” regarding nurse infections, while during the COVID-19 period, news reports focused on topics such as “group infection,” “duty,” “contact,” “working environment,” “site,” and “rest.”

### Theme 4. Roles and Responsibilities of Nursing Professionals

The topic “roles and responsibilities of nursing professionals,” which was the lowest during the SARS period, showed an upward trend from the influenza A period to the COVID-19 period, and during the COVID-19 period, it had the largest change among the 6 derived topics.

The keywords of the “roles and responsibilities of nursing professionals” topic changed mainly based on specific incidents reflecting the situation at the time. During the SARS period, specific incidents related to keywords such as “blood,” “drunk driving,” “hospital,” and “sexual harassment” included a case in which a nurse switched the blood of a drunk driving colleague with her own blood and submitted it, a case in which a doctor sexually harassed a nurse, and an illegal medical practice by a nurse or nursing assistant. During the influenza A period, keywords related to the incidents and accidents at the time, such as a case in which a newborn was switched due to a nurse’s mistake, included “adjudge” and “obstetrics and gynecology” and related to bioethics, such as euthanasia, included “grandmother” and “death with dignity.” During the MERS period, keywords such as “family,” “thought,” “heart,” “gratitude,” and “cheer” showed support for doctors and nurses who were infected during the MERS response process, which occurred mainly in specific hospitals. During the COVID-19 period, keywords such as “medical school quota,” “expansion,” “Korean Medical Association,” and “Korean Nurses Association” mainly focused on issues such as doctors’ strikes and nurses who stayed by patients’ sides during the infectious disease outbreak.

### Theme 5-7. Topics Derived Only During a Particular Infectious Disease Period

The fifth topic “government response and public opinion” was derived during the MERS and COVID-19 periods, and it had the second lowest proportion in both periods. During the MERS period, from the keywords “virus,” “the public,” “outbreak,” “anxiety,” “information,” “fear,” and “open,” we could infer that the public experienced anxiety due to a lack of information on new infectious diseases from the government’s disclosure of information. During the COVID-19 period, keywords such as “nurse,” “president,” “gratitude,” “encouragement,” “controversy,” and “criticism” emphasized the controversy over the president’s thank you message to nurses during the doctors’ strike and the public’s support for the struggles of medical staff, such as the “Thanks to Challenge,” as social issues.

“Vaccination for emerging infectious diseases” was derived only during the influenza A and COVID-19 periods, and this topic had the lowest proportion of all topics in both periods. During the influenza A period, the keywords “student,” “school,” and “school parent” indicate that children and adolescents were the main targets of vaccination, while during the COVID-19 period, the keywords “nursing facility,” “targets,” and “worker” show that the targets of vaccination changed to older adults and health care workers. “AstraZeneca,” “side effects,” “Pfizer,” and “effect,” which appeared only during the COVID-19 period, indicate the situation of and public interest in the development of COVID-19 vaccines, which were not available at the beginning of the outbreak, and vaccination.

The main keywords of the “response to emerging infectious diseases in other countries” topic, which was derived only during the SARS period, consisted of the names of countries that experienced significant damage from new infectious diseases, such as “China,” “Taiwan,” and “Hong Kong,” and keywords about the situation of the outbreak, such as “death,” “outbreak,” “addition,” and “transmission.”

## Discussion

### Principal Findings

This study was based on the basic premise that, in public health crises such as new infectious diseases, media reports related to the field, including nursing and nurses, become more active, reflecting high social interest in major issues. This study attempted to explore the problems that were highlighted during 4 new infectious diseases that occurred in Korea and considerations regarding social awareness and improvement to policies in relation to major nursing issues. Therefore, this study compared and analyzed the main agenda and potential meaning of news articles on nursing and nurses for each new infectious disease period using the big data analysis techniques of topic modeling and keyword network analysis. In addition, we discussed the understanding of current nursing phenomena and the trends and implications of public opinion surrounding nursing based on the results.

As a result of topic modeling analysis, 7 topics were classified from all news articles. The commonly derived topics from the 4 infectious disease periods included “demand for nurses,” “response to emerging infectious diseases in Korea,” “vulnerability in the working environment,” and “roles and responsibilities of nursing professionals.” Topics with the same higher concept had changes in the associated keywords according to the infectious disease period. The following sections discuss the potential meaning in each topic by referring to the visualization results of the related keyword network map, focusing on the topics commonly derived during the new infectious disease periods.

### Response to Emerging Infectious Diseases in Korea

First, the keywords for the topics during the SARS, MERS, influenza A, and COVID-19 periods showed similar patterns when analyzing the relevance of the topic by period. Depending on the duration of the infectious disease, the keywords “outbreak,” “spread,” “addition,” and “pandemic” were used to express the seriousness of the domestic infectious disease situation. The potential meaning of the words related to the keywords “National Institute of Health,” “dedication,” and “designation” during SARS and “hub-hospital” during influenza A was the government’s practical policy proposal to prepare for new infectious diseases. These policies included the designation of regional hub hospitals and securing state-designated inpatient treatment beds that were proposed during SARS and influenza A and were implemented and partially improved after the end of those infectious disease periods. However, the number of public medical resources, such as treatment beds and health professionals, that could be practically used was significantly insufficient for the number of infected patients during the MERS and COVID-19 periods [24].

In addition, keywords such as “medical staff,” “infection,” “closed,” “hospital room,” and “hospitalization” during MERS and COVID-19 highlighted vulnerabilities in emergency room environments, including inadequate infection control measures and gaps in infection control in nursing hospitals and nursing facilities where older adult patients, who are vulnerable to infection, are mainly hospitalized. The emergency room is where infected patients primarily access the hospital. Problems including the lack of facilities such as separate isolation zones and negative pressure isolation rooms for the treatment of infected patients revealed during the MERS period, the lack of education on infection control, and the response of emergency medical workers increase the risk of medical staff becoming infected and are viciously linked to patient infection and the lack of response personnel [25]. In addition, in emergency rooms, there is a greater need for systematic infection control by professional personnel such as nurses because older adult patients in nursing hospitals and facilities are more likely to contract infectious diseases due to low immunity and group living and the risk of death from infection is high [26,27]. This is a matter to be considered first in terms of policy for infection control in environments where patients and medical personnel are at a high risk of infection. After MERS, the enforcement regulations outlined in the Emergency Medical Service Act were revised to improve emergency room facilities and manpower standards [28], and through the revision of the enforcement regulations of the Medical Act during the COVID-19 period, the standards and fees for mandatory infection control rooms for infection control were expanded [29]. However, the effectiveness of the policy is still limited because it does not resolve gaps in infection control outside the scope of the policy, such as the lack of infection control experts and nursing hospitals and facilities with fewer than 100 beds. Although policies such as the revision of the Emergency Medical Service Act aimed to address these gaps, their effectiveness remains limited due to insufficient implementation and resource allocation. Similar challenges in emergency room preparedness were noted in countries such as Italy and the United States during the COVID-19 pandemic, underscoring the global nature of these vulnerabilities.

### Demand for Nurses

In the “demand for nurses” topic, the need to increase the number of nurses has been steadily highlighted over the past 20 years, and the keyword “insufficiency,” which appeared only during the influenza A and COVID-19 periods shows that the shortage of nurses during the COVID-19 period was also a problem that was raised during the influenza A period. In the topic analysis results, the focus on nursing demand changed from health and welfare services to responding to infectious diseases, and in the change of keywords over time, the area of public interest also changed according to the reason for the demand in nurses and the field of activity. The areas where nurses’ activities are performed are diverse and include medical institutions, communities, schools, and businesses; nurses are also recognized as particularly indispensable for the national welfare service, especially in a society where the number of older adult and single-person households is increasing [30,31]. Among the government’s systems related to the demand for nurses, the comprehensive nursing service (currently, the integrated nursing care service), which was introduced in 2013 to reduce the burden of care and expenses for people in a nuclear family and an aging society, accelerated the expansion of the service, as domestic nursing and door-to-door cultural issues were raised in terms of hospital infection control during the MERS period in 2015. Nurses with specialized knowledge and skills were recognized as a major element of the service, which proceeded to actively use nurses [32,33]. Many previous studies on integrated nursing care services have shown positive effects of these services for improving patient safety, reducing hospital infections, and increasing patient satisfaction with medical services [32,34,35]. Accordingly, there seems to be no difference in public opinion on the need for a change in the demographic and social structure, the response to infectious disasters at the health care level, a quantitative increase in nurse demand and quantity, and policies to increase the number nurses.

In terms of meeting the demand for nursing during periods of new infectious diseases, the focus was on recruiting nurses and distributing manpower to prevent the infection of the vulnerable, such as older adults, and school quarantine during the SARS and influenza A periods. Changes for the issue of nursing supply were mainly made to comprehensive nursing services as a preventive measure against hospital infections. During the COVID-19 period, there was a shortage of nursing personnel who could be immediately placed in response to infectious diseases in medical institutions in nonmetropolitan areas such as Daegu, life treatment centers, and hospitals dedicated to infectious diseases. The government filled the demand for nurses with temporary measures through the dispatch of nursing personnel recruited through the input of nursing officers and allowances. However, the urgent recruitment of the personnel needed in the field of infectious diseases was ineffective due to insufficient nursing experience, education, and training [36,37]. It is believed that the gap in the nursing field continues because there is a real need for nurses with the experience and capacity to care for infectious disease patients and intensive care patients, while the government’s policies for and public opinion about increasing the nursing supply focused purely on the number of nurses rather than the quality and capacity of nursing.

The fact that the policy to increase the number of nurses recognized nursing as a job that not only resolves the imbalance of local medical personnel or meets medical needs but also resolves employment difficulties and guarantees employment after graduation is similar to the ideas expressed in the original news article [38] titled “Graduation is Employment, Nursing and Explosion of Popularity” and research showing that high school students who are about to pursue careers perceive nursing as an economically stable and good job compared with other occupations [39]. In addition, in research analyzing the meaning of work for new nurses in 2021, more than 50% of the nurses had family and relatives who recommended they pursue nursing, or they applied to the nursing department because they were able to easily get a job after graduation [40].

Since 2008, the year just before the influenza A outbreak, Korea implemented a policy to establish a nursing department and increase the number of admissions. As a result of this policy, during the swine flu period, there were 414,000 licensed nurses in 2019, and 20,000 new nurses entered the workforce every year [30]. However, there are only 215,000 nurses working in medical and health institutions, which represents about one-half of the licensed nurses [41]. According to data from a survey on the status of hospital nursing staff placements every year since 2010, the turnover of new nurses increased from 30.5% in 2011 to 47.7% in 2020, and the number one reason for resignation within a year after the announcement was “work maladjustment” [42,43]. The burden of work on new nurses was correlated with the reality shock caused by the conflict between the hospital organizational culture and nursing professionals [44]. Nurses are aware of the risk of infection at infectious disease sites, but they have a sense of calling and must perform patient care at the forefront [45,46]. In patient care, the experience of a conflict between infection risk situations and professional beliefs increased nurses’ intentions to leave more than the general situation of care [10,47]. Based on these preceding studies, there is likely a difference between the actual clinical field and the public’s perception of nursing in terms of the purpose of employment identified in the results of this study. Although economic feasibility cannot be excluded from policies to resolve employment difficulties, it is necessary to consider nursing professionals in policy and media in order to prevent the departure of new nurses, increase the number of experienced nurses, and change the public’s perception of nurses.

### Vulnerability in the Working Environment

In the “demand for nurses” topic during the influenza A and COVID-19 outbreaks, a critical issue was the shortage of skilled nurses—particularly in nonmetropolitan areas—in addition to skills to handle special equipment such as ventilators and extracorporeal membrane oxygen supply required for intensive care nursing as well as clinical experience [36]. Given the strong link between nursing demand, supply, and working conditions, the topics “demand for nurses” and “vulnerability in the working environment” are closely interconnected [37] and can be interpreted together. In this study, hot topics included conflicts between women’s work-family balance during the SARS period, health risks due to night work, domestic medical facilities and manpower at a lower level than in OECD countries during the influenza A period, and the shortage of nurses in nonmetropolitan areas. The changes in keywords such as high secondary infections of nurses related to new infections, emergency rooms, and nursing hospitals; work weighting; and society’s expectations for sacrifice and commitment should be considered as major variables to resolve the shortage of nurses.

According to a survey by the Hospital Nurses Association, the average resignation rate of domestic nurses over the past decade has been over 13%. When the reasons for resignation in 2010 and 2020 were compared, the second highest reason of “marriage, childbirth, and childcare” in 2010 fell significantly from 14.8% of responses in 2010 to 6.3% of responses in 2020. “Work maladjustment,” which was ranked third (13.1%) in 2010, was ranked first (17.1%) in 2020, and “transition to other jobs,” which was ranked second (5.9%) in 2010, increased to 12.2% in 2020 [42,43]. This is likely the result of government policy and efforts to improve the conflict that comes from the balance between work and family for female nurses, which appeared in the topic during SARS. However, high-risk working environments for infection and the lack of experienced nurses during MERS and COVID-19, during which there was a focus on the quantitative increase of nurses following the influenza period; the policy to increase the number of nurses; and the poor treatment suggest that more active interventions and improvement are needed at the organizational level.

Inadequate facilities in which to rest and the social stigma of being perceived as infection carriers—both personally and for family members—accelerate burnout and increase turnover intentions among nurses, and secondary infections among nurses create a vicious cycle of labor shortages and increased patient infections [48,49]. The government expanded the admission quota for nursing colleges starting in 2008 in an effort to increase the absolute total number of nurses, but it did not have a significant impact on resolving the shortage of nurses in local small and medium-sized hospitals [50]. In addition, in March 2018, the Ministry of Health and Welfare announced policies aimed at improving nurses’ working conditions, addressing human rights violations such as workplace bullying and sexual harassment, expanding nursing personnel, strengthening professionalism, improving the quality of nursing services, and creating a policy foundation for nursing personnel [51]. In this regard, the system for calculating inpatient nursing management fees was revised to base payments on patient-to-nurse ratios according to the actual situation in local hospitals in April 2018. The system was improved so that additional profits from hospitalization fees generated by changes based on the number of patients were used to improve the treatment of nurses. In addition, the night-only nurse management fee and the night-care fee for general wards established in October 2019 were applied to general hospitals and hospitals outside of Seoul so that additional allowances for night work could be paid. However, since both systems are merely recommendations, there were practical limitations for nurses to feel that their treatment was improved [52]. In February 2019, a Task Force Team was promoted to oversee nursing policies such as managing the supply and demand of nursing personnel and improving the working environment. In May 2021, a nursing policy department was established in the Ministry of Health and Welfare with social consensus on the need for a dedicated department during the COVID-19 period [42]. As such, the government has continuously implemented policies to address the shortage of nurses, and evaluation of the effectiveness of some policies is expected to take some time. This is an opportune time to reconsider whether fragmented and temporary approaches to nursing challenges have exacerbated systemic issues, such as the revision of the medical law that tried to reorganize nursing assistants from the “demand for nurses” topic during the MERS period or the “vulnerability in the working environment” topic, which is related to a high risk of infectious diseases and the need for high-intensity work. In addition, active monitoring is needed to determine how closely a series of policies has approached the practical problems of the nursing field, and feedback mechanisms during a changing infectious disease situation are needed. Although these policies mark progress, their voluntary nature limits their effectiveness in achieving widespread improvements.

### Roles and Responsibilities of Nursing Professionals

The keyword changes in the topic of “roles and responsibilities of nursing professionals” mainly included incidents and accidents related to the morality of nurses, such as drunk driving and illegal medical practices during the SARS and influenza A periods. Given the focus on keywords related to infectious diseases during the MERS and COVID-19 periods, the conflict over work ethics in the nursing of infected patients versus the nurse’s own safety and social expectations was considered a major agenda.

Since the image of nurses formed in the media has been considered to influence potential future nurses’ career choices and intentions and some stories about nurses can negatively influence not only nursing supply but also potential improvements in the working environment and treatment of nurses, many previous studies have proposed monitoring of the images of nursing expressed in the media and internal management of the nursing community [3,53].

The analysis of correlations between keywords during the MERS period highlighted social expectations for nurses in special situations such as public health disasters as well as conflicts between nurses’ protective instincts and work ethics and their families and colleagues. In this regard, the original news articles reviewed in this study described the critical condition of medical personnel who had secondary MERS infections and experiences with social isolation, such as nurses’ children being ostracized by their neighbors due to the stigma of working with MERS-infected patients [54]. The ambivalence of social expectations for nurses responding to infectious diseases is reflected by the description of medical personnel as heroes through the “Thanks to the Challenge” campaign and the praise for volunteer nurses in Daegu and Gyeongbuk. The precedent of the government’s legal response to the collective resignation of Taiwanese medical staff due to the infection and death of medical personnel is another example and was captured in the topic “response to emerging infectious diseases in other countries” during SARS. The ambivalence was also present in an overseas example [3] that described a nurse volunteering at an infectious disease site during the Ebola outbreak as a virus eradication hero versus an infection spreader and villain. Since social stigma and isolation during an infectious disease outbreak add to the mental and social stress of nurses and influence nursing and turnover intentions, the following are needed: accurate information delivery by the media, a stable medical system, and an organizational protection and support system [48,49].

Meanwhile, in the context of the COVID-19 period, the government attempted to implement a policy to expand the number of medical schools to respond to infectious diseases, and the related medical strike led to the aggravation of nurses’ work [55]. Previous studies have highlighted the 2-sided phenomenon of social expectations, which consists of systemic problems such as poor hospital infection control, inadequate government response, and increased public fear through negative media reports rather than the attitude of medical personnel, including nurses, when responding to infectious diseases [6,48]. This is similar to the relationships between the topics of “government response and public opinion” and “roles and responsibilities of nursing professionals,” which include indiscriminate disclosure of information about infectious diseases in the media during the MERS period and the controversy over the president’s thank you message to nurses during a doctor strike during the COVID-19 period. This suggests that active interest and the adoption of a leading role by the government are needed to avoid delays in policy and social consensus as a result of insufficient government responses and issue-oriented media reports.

### Limitations

In this study, limitations were related to the search used to identify issues in the nursing field as well as the data collection period, which was restricted to the periods around outbreaks of new infectious diseases and limited the ability to compare the situation before and after an outbreak. Therefore, future research should adopt a multifaceted approach that incorporates time series analysis, dynamic topic modeling, and expanded data collection periods to capture broader trends. In addition, as this study started during the COVID-19 outbreak and we were unable to predict when the outbreak would end, we collected data only until May 31, 2021, due to limited time available for data analysis. Therefore, any changes in the situation after that data were not reflected in the research results, and we recommend collecting and analyzing data after that date.

### Conclusion

This study analyzed the relevance of nursing-related agendas in news articles during 4 major EID outbreaks. Our analysis revealed that nurse shortages, poor working conditions, and inadequate treatment of nurses were recurring issues across the epidemics and were not unique to the COVID-19 pandemic. Although society and nursing professionals agree on the essential role of nurses in the health sector, significant differences persist in perceptions of their roles and responsibilities. The results suggest that retaining experienced nurses to strengthen crisis response capabilities should be closely linked to the working environment. Addressing nurse shortages requires not only increasing their numbers but also implementing substantial policy support to ensure safe working environments, better treatment conditions, and heightened societal recognition of their roles. Medical organizations, professional associations, governments, and the media should develop policies that incorporate nurses’ perspectives. These efforts can foster positive social consensus on nursing and support a stable health care system during health crises. Our findings are expected to be used as a basis for establishing strategies to improve the professional role, environment, and treatment of nurses during health crisis situations. Finally, these findings emphasize the need for holistic reforms to strengthen nursing’s role in mitigating future health crises. We recommend further research on the effects and changes of nursing-related policies.

## Abbreviations

EID: emerging infectious disease
LDA: latent Dirichlet allocation
MERS: Middle East respiratory syndrome
OECD: Organisation for Economic Co-operation and Development
SARS: severe acute respiratory syndrome
WHO: World Health Organization
